# Robotic transthoracic approach to ectopic mediastinal parathyroid adenoma: A case report

**DOI:** 10.1016/j.ijscr.2024.110719

**Published:** 2024-12-09

**Authors:** Gavin P. Dowling, Cian M. Hehir, Gordon R. Daly, Maher N. Shuhaibar, Peter Walshe

**Affiliations:** a Department of Surgery, Royal College of Surgeons in Ireland (RCSI) University of Medicine and Health Sciences, Dublin, Ireland; b Department of Surgery, Beaumont Hospital, Dublin, Ireland; c Department of Surgery, Mater Misericordiae University Hospital, Dublin, Ireland

**Keywords:** Parathyroid adenoma, Parathyroidectomy, Robotic surgery, Mediastinum, Case report

## Abstract

**Introduction:**

Ectopic parathyroid adenomas represent an important cause of refractory hyperparathyroidism. While most ectopic mediastinal parathyroid adenomas can be accessed through a transcervical approach, this is not always feasible, posing a significant challenge.

**Case presentation:**

We report the case of a 60-year-old female patient who presented with symptomatic hyperparathyroidism. Sestamibi scan was performed and failed to identify a parathyroid adenoma. Bilateral neck exploration was performed on twice, with normal parathyroid glands excised on both occasions. At this stage, a repeat sestamibi and CT scan revealed a suspected parathyroid adenoma in the mediastinum. A transthoracic robotic parathyroidectomy was performed, and the adenoma successfully excised. A full serological and symptomatic recovery was achieved.

**Discussion:**

Mediastinal parathyroid adenomas can pose significant diagnostic and treatment challenges.

**Conclusion:**

A robotic transthoracic approach demonstrates a safe method for the removal of parathyroid adenomas which are inaccessible transcervically, with low morbidity to patients.

## Highlights

• Ectopic mediastinal parathyroid adenomas represent a rare cause of refractory hypercalcaemia.
• Most mediastinal parathyroid adenomas can be accessed via a transcervical approach.
• Transthoracic access is required in certain cases where the gland is too low to be accessed via neck incision.
• A robotic transthoracic approach to these lesions should be considered to avoid the significant morbidity associated with sternotomy or thoracotomy.

## 1 Introduction

Primary hyperparathyroidism (pHPT) is characterised by excessive secretion of parathyroid hormone (PTH), resulting in serum hypercalcaemia [1]. This is most often caused by a parathyroid adenoma (90 %), and less frequently by parathyroid hyperplasia (6 %) or parathyroid carcinoma (<1 %) [2]. Asymptomatic presentation of this endocrine disorder is not uncommon, with incidental serum hypercalcaemia being the only finding. However common clinical manifestations include osteoporosis, nephrolithiasis, lethargy and nausea [3]. Surgical excision of the parathyroid adenoma remains the mainstay of treatment. This can be performed with bilateral neck exploration, or now more commonly through minimally invasive techniques, due to improvements in localisation studies. While inadequate excision, development of a second adenoma or cancer remains causes of refractory pHPT, ectopic parathyroid adenomas must be considered as a potential cause. Ectopic parathyroid glands are present in approximately 15 % of patients, with mediastinal parathyroid adenomas occurring in up to 20 % of these patients [4,5]. Many of these mediastinal adenomas can be successfully excised through a standard cervical excision [6]. However, in cases where this is not feasible, a median sternotomy may be required, associated with significant post-operative morbidity [7]. Significant improvements in localisation techniques have led to many surgeons favouring less invasive options for mediastinal exploration [8,9]. Herein, we present a case of pHPT due to a mediastinal parathyroid adenoma, located below the manubriosternal angle.

## 2 Presentation of case

A 60-year-old female presented with a 3-month history of renal stones, depression and nausea. Past medical history was otherwise unremarkable, and no previous intervention was performed. On investigation, serum adjusted calcium (2.8 mmol/l) and serum parathyroid hormone (102 pg/ml) were significantly elevated. A parathyroid adenoma was suspected as the cause for this symptomatic hyperparathyroidism, and the patient underwent a sestamibi scan, which failed to identify a parathyroid adenoma. Notwithstanding this, a bilateral neck exploration was performed and a possible enlarged parathyroid adenoma was removed from the left side of the neck. Despite this, the PTH and calcium remained raised and the patient symptoms persisted. Subsequent exploration of the neck was undertaken, and a right lower parathyroid was excised. Once again, this failed to cure the symptoms and serum calcium and PTH remained elevated.

**Fig. 1:**
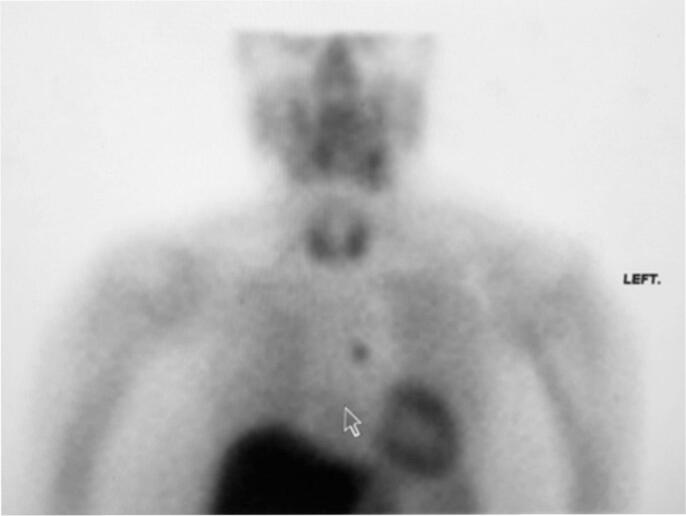
Sestamibi scan demonstrating increased uptake in the mediastinum.

**Fig. 2:**
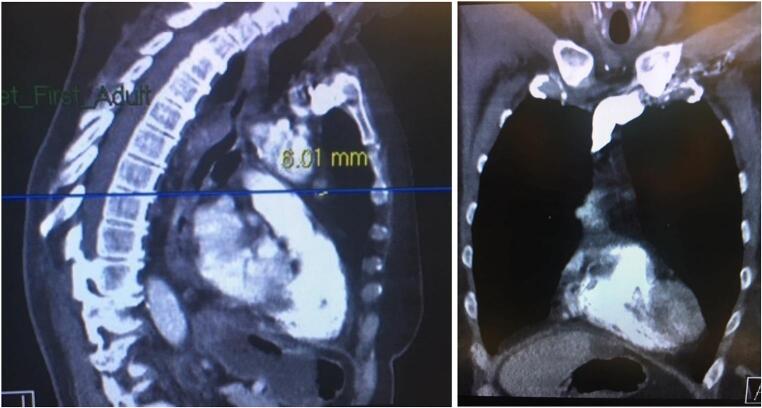
CT scan showing mass in the anterior mediastinum, below the angle of Louis.

The case was discussed by the multidisciplinary team, and the patient was considered for treatment with cinacalcet. A sestamibi scan was repeated at this stage, revealing increased uptake of the tracer in the mediastinum (Fig. 1). A contrast-enhanced CT scan of the neck and thorax identified a corresponding mass, a suspected parathyroid adenoma (Fig. 2). The parathyroid was located below the angle of Louis, at the level of the mid-point of the arch of the aorta on the left-hand side. In light of this new finding of a parathyroid adenoma, surgical excision was preferred over cinacalcet treatment.

**Fig. 3:**
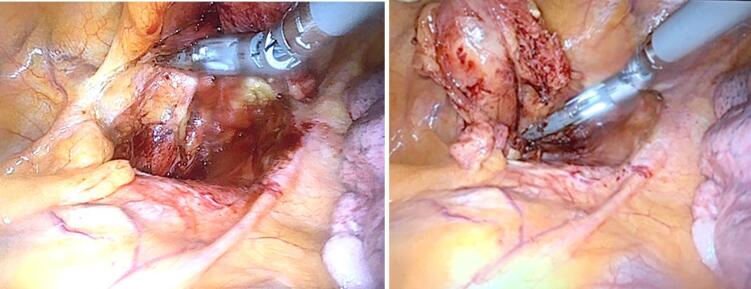
Intra-operative images of the ectopic mediastinal parathyroid adenoma from the DaVinci robot.

**Fig. 4:**
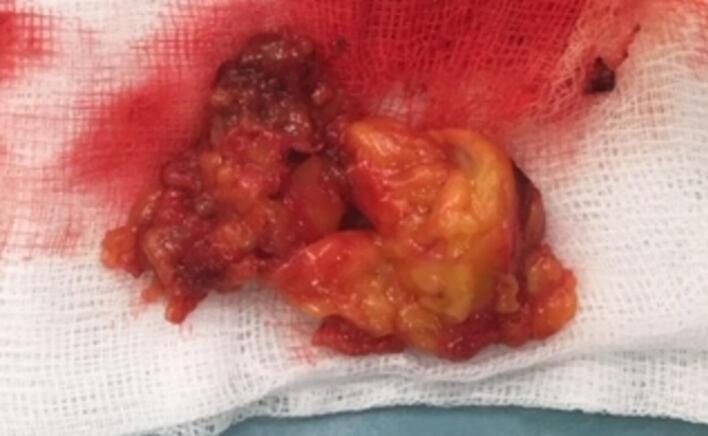
Excised parathyroid adenoma.

The patient underwent transthoracic robotic parathyroidectomy, using the DaVinci robot, and a parathyroid adenoma was successfully identified and removed, while the phrenic nerve and adjacent structures were preserved fully (Fig. 3, Fig. 4). This was confirmed as parathyroid adenoma on pathological examination. Post-operatively, the patient suffered from symptomatic hypocalcaemia due to hungry bone syndrome, which resolved over the period of a week. Serological and symptomatic recovery was achieved and the patient was discharged without complication and remains well 6 months post-operatively.

## 3 Discussion

This case describes an approach to robotic transthoracic mediastinal parathyroidectomy, which removes the need for a formal sternotomy. Variation exists in the definition of mediastinal parathyroid adenomas among studies, resulting in disparities between reported incidence rates [4,10]. Definitions vary between being located below the clavicle to, in older studies, necessitating a median sternotomy [9,11]. Deciding which cases are unsuitable for a cervical approach, and thus requiring a thoracic approach, poses a significant clinical challenge. One study reported that the majority of glands located ≥6 cm below the superior aspect of the clavicle will likely require a thoracic approach [12]. The manubriosternal angle (angle of Louis) is located approximately this distance from the jugular notch and can thus be a useful guide in deciding the optimal surgical approach [13].

Preoperative localisation of the mediastinal parathyroid adenoma is vitally important to ensure successful surgical excision. As was the case in this instance, many ectopic parathyroid adenomas will only be diagnosed following a non-curative bilateral neck exploration, prompting further investigation. Repeated failed neck explorations are associated with an increased risk of post-operative permanent hypoparathyroidism, and thus parathyroid re-implantation should also be considered as appropriate [14]. In this case report, the reason the first sestamibi was negative was due to competing parathyroids for a given amount of sestamibi radioiodine. Following the removal of the prior 2 normal parathyroids there was less PTH to compete, and subsequently the parathyroid adenoma was identified.

In an attempt to avoid the morbidity of a median sternotomy or thoracotomy, less invasive techniques for mediastinal exploration have been adopted. Video-assisted thoracoscopy (VATS) is one such minimally invasive approach that has gained widespread adoption [6,15]. Transthoracic robot-assisted mediastinal parathyroidectomy is a relatively novel technique which has shown promise as a safe and effective approach to mediastinal parathyroid adenomas that are inaccessible from the neck [14,16,17].

## 4 Conclusion

This case demonstrates a novel, safe and established approach for the removal of parathyroid adenomas which are below the angle of Louis associated with significantly less morbidity to patients.

## 5 Methods

This case report was reported in line with the SCARE criteria [18].

## Consent

Written informed consent was obtained from the patient for publication and any accompanying images. A copy of the written consent is available for review by the Editor-in-Chief of this journal on request.

## Ethical approval

Case reports are exempt from ethical approval is by the Beaumont Hospital Ethics (Medical Research) Committee in our institution.

## Guarantor

Peter Walshe

## Research registration number

NA

## Funding

This report did not receive any form of funding from any funding agencies in the public, commercial, or not-for-profit sectors.

## Author contribution

GPD – acquisition of data, data analysis, writing – original draft, Writing – review & editing, conceptualization.

CH, GRD - Writing – review & editing.

MNS - acquisition of data, conceptualization.

PW - acquisition of data, data analysis, writing – review & editing, conceptualization, supervision.

## Conflict of interest statement

The authors have declared that no conflicts of interest exist. This research did not receive any specific grant from funding agencies in the public, commercial, or not-for-profit sectors.
